# Effects of Residual Anterior Lens Epithelial Cell Removal on Axial Position of Intraocular Lens after Cataract Surgery

**DOI:** 10.1155/2018/9704892

**Published:** 2018-08-19

**Authors:** Seung Pil Bang, Young-Sik Yoo, Jong Hwa Jun, Choun-Ki Joo

**Affiliations:** ^1^Department of Ophthalmology, Dongsan Medical Center, Keimyung University School of Medicine, Daegu, Republic of Korea; ^2^Catholic Institute for Visual Science, The Catholic University of Korea, Seoul, Republic of Korea; ^3^Department of Ophthalmology and Visual Science, Seoul St. Mary's Hospital, College of Medicine, The Catholic University of Korea, Seoul, Republic of Korea

## Abstract

**Purpose:**

The aim of this study was to assess the effects of residual anterior lens epithelial cell (LEC) removal by anterior capsule polishing on the effective lens position (ELP) and axial position stability of the intraocular lens (IOL) after cataract surgery via postoperative measurement of the anterior chamber depth.

**Methods:**

We enrolled 30 patients (60 eyes) requiring bilateral cataract surgery for age-related cataracts. Meticulous anterior capsule polishing and removal of residual LECs under the capsule were performed using a bimanual irrigation/aspiration system for one randomly selected eye in each patient. The eye without polishing served as a control. ELP was measured at five different time points after surgery, and axial shifting of IOL was determined at each visit by comparison with the position at the previous visit.

**Results:**

The polishing and control groups showed significant differences with regard to the mean ELP at 1 (3.40 ± 0.29 versus 3.53 ± 0.32 mm, resp.; *p*=0.026) and 2 months (3.42 ± 0.32 versus 3.61 ± 0.35 mm, resp.; *p*=0.001) after surgery, the mean standard deviation for the five ELP values (0.087 ± 0.093 versus 0.159 ± 0.138 mm, *p*=0.001), and the root mean square of the change in ELP at each follow-up visit (0.124 ± 0.034 versus 0.246 ± 0.038 mm, *p*=0.047). The eyes in the control group exhibited a tendency for backward IOL movement with a concurrent hyperopic shift in refraction of approximately 0.2 diopter at 2 months after surgery.

**Conclusion:**

Our findings suggest that residual anterior LEC polishing enhances the axial position stability of IOLs, without any complications, after cataract surgery.

## 1. Introduction

In recent years, phacoemulsification with concurrent foldable intraocular lens (IOL) implantation has taken the place of refractive surgery, with the development and increase in the popularity of various refraction-correcting IOLs, including multifocal or toric IOLs. In addition to an accurate IOL power calculation formula and ocular biometry, the precise determination of the effective lens position (ELP) is essential to optimize the postoperative refractive outcomes [1, 2]. ELP, described as the distance between the anterior surface of the cornea and the IOL plane, indicates the axial position of IOL [3]. Forward movement of IOL from the estimated ELP results in myopia, while backward displacement leads to a hyperopic shift in refraction [4, 5]. One study reported that inaccurate ELP prediction can account for 22% to 38% of the total refractive prediction error [6], while a postoperative shift in ELP could induce an unexpected refractive change apart from the prediction error. The reciprocal action between capsular fibrosis and bag fusion possibly accounts for the change in ELP after surgery [7].

Residual lens epithelial cells (LECs) after cataract surgery play a significant role in the development and progression of capsule fibrosis and contraction [8–10]. Several studies have reported that the removal of residual anterior LECs resulted in delayed or lesser capsular bag contraction and anterior capsule fibrosis [11–14]. We speculated that the removal of residual anterior LECs by anterior capsule polishing may minimize changes in ELP and the axial position of IOL induced by capsular bag contraction and anterior capsule fibrosis. Accordingly, we designed the present study to evaluate changes in ELP in order to identify the axial position stability of IOL and associated refractive alterations after cataract surgery with anterior capsule polishing.

## 2. Patients and Methods

### 2.1. Patients

We enrolled 30 patients (60 eyes) from January 2016 to April 2017. The research was performed at the Department of Ophthalmology in Dongsan Medical Center, which is affiliated with Keimyung University in Daegu, Republic of Korea. All studies and measurements were in accordance with the tenets of the Declaration of Helsinki, and the study protocol was reviewed and approved by the Ethics Committee of Dongsan Medical Center (Approval no. 2016-01-001). Informed consent for participation was obtained from all patients. The inclusion criteria were as follows: bilateral age-related cataract with a favorable clinical status and uneventful in-the-bag IOL implantation in both eyes. The exclusion criteria were as follows: a history of intraocular surgery or corneal laser surgery, a history of ocular trauma or uveitis, severe fundus pathology, an axial length (AL) of <22.0 mm or >24.0 mm, pseudoexfoliation syndrome (PEX), poor pupil dilation, zonular weakening or tension ring insertion, too large or too small continuous curvilinear capsulorhexis (CCC), eccentric CCC, radial tear in the anterior capsule, and inability to attend follow-up appointments on time.

### 2.2. Cataract Classification

Before surgery, the opacity in each eye was evaluated using the Lens Opacities Classification System III (LOCS III) [15] after pupil dilation with 0.5% tropicamide/0.5% phenylephrine fixed combination eye drops (Tropherine®, Hanmi Pharm, Seoul, Korea). A cataract surgery specialist graded every eye using slit-lamp examination for matching nuclear opalescence and color, cortical cataract, and posterior subcapsular cataract with opacities on standardized color photographs. Patients with a large discrepancy in the severity of cataract between the right and left eyes, those with brunescent or mature cataract, and those with anterior subcapsular opacity were excluded. Nuclear opalescence, cortical cataract, and posterior subcapsular cataract grades were used to confirm similarities between the polishing and control groups in terms of the cataract grade and evaluate the effect of cortical cataract on capsular contraction and fibrosis after surgery.

### 2.3. Surgical Procedure

All study patients underwent the necessary laboratory tests and clinical examinations. The IOL power was set to achieve postoperative refraction between +0.25 and −0.25 diopter (D) using optical low-coherence reflectometry (Lenstar LS900®, Haag-Streit AG, Bern, Switzerland). The preoperative best-corrected visual acuity (BCVA) was confirmed and converted to logMAR units. The anterior chamber depth (ACD) was measured before surgery using A-scan (AXIS-II PR®, Quantel Medical Inc., Paris, France). Approximately 30 min before surgery, 0.5% tropicamide/0.5% phenylephrine fixed combination eye drops (Tropherine®, Hanmi Pharm) were instilled in the patients' eyes twice within 5 min for maximal pupil dilation. A single surgeon (JHJ) performed all surgeries with a 2.85 mm coaxial incision (Infiniti® vision system, Alcon Laboratories, Fort Worth, TX, USA). After topical anesthesia with 0.5% proparacaine eye drops (Paracaine®, Hanmi Pharm), a clear corneal incision was placed at the 9 o'clock (right eye) and 2 o'clock (left eye) positions. A centered CCC with a 5.5 mm diameter was prepared using capsulorhexis forceps. For the precise achievement of an equal CCC size, we used a 6 mm diameter capsulorhexis marker (K3‐7850, Katena, Denville, NJ, USA) for a 5.5 mm diameter CCC; too large, too small, or eccentric CCCs were excluded. Subsequently, thorough hydrodissection was performed to freely rotate the nucleus, following which phacochop nucleofractis was used for emulsification and removal of the nucleus.

**Figure 1:**
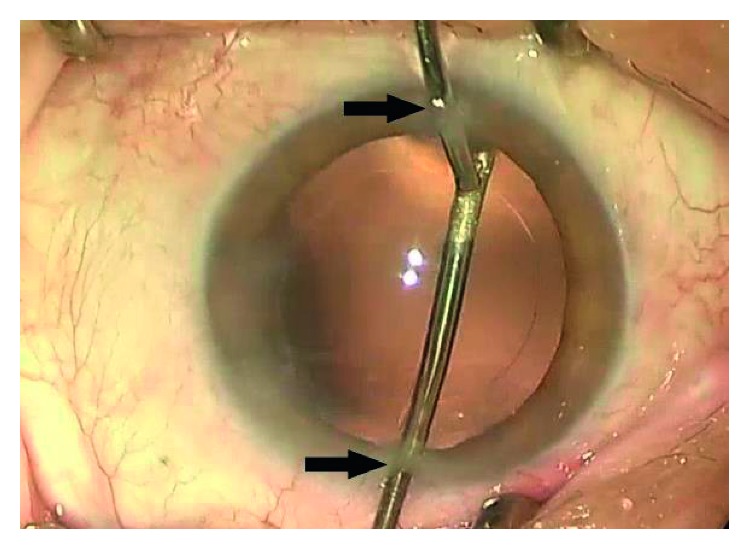
Residual anterior lens epithelial cell (LEC) removal using a bimanual irrigation/aspiration system during cataract surgery. Accessibility to the entire capsule is achieved by the creation of two paracenteses (arrows) at a distance of 180° from each other. Complete polishing of the anterior capsule is performed for the removal of all visible LECs under the capsule.

To eliminate any bias, the decision to perform anterior polishing or not was made only after the completion of cortex aspiration using an ordinary one-hand irrigation/aspiration system, without reference to the amount of visible residual LECs. Subsequently, the eyes were assigned to a polishing or control group using a table of random numbers generated by Microsoft Excel for Windows 2013, according to Monte Carlo calculations. For the control group eyes, no additional anterior capsular polishing was performed after routine cortex aspiration. For the polishing group eyes, residual LECs in the anterior capsule were aspirated using a bimanual irrigation/aspiration system (Figure 1). Accessibility to the entire capsule was achieved by the creation of two paracenteses at a distance of 180° from each other. Anterior capsule polishing was performed for the removal of all visible LECs. Following insertion of a hydrophobic one-piece IOL (Sensar® AAB00, Abbott Medical Optics Inc., Santa Ana, CA, USA) and aspiration of the ophthalmic viscosurgical device, the corneal wound was hydrated. Patients were treated with 0.5% moxifloxacin eye drops (Vigamox®, Alcon Laboratories) and 1% prednisolone acetate ophthalmic suspension (Pred Forte®, Allergan, Irvine, CA, USA) every 2 h for 3 days after surgery, following which the frequency was tapered to four times a day over 3 weeks or as clinically indicated.

### 2.4. ELP Measurements

ELP was defined as the distance from the anterior surface of the cornea to the anterior surface of IOL in the pupil center, along the optical axis. It was measured on an A-scan at 1 day, 3 days, 1 week, 1 month, and 2 months after surgery. Comparison of the mean ELP was considered inappropriate because forward and backward movements could be partly neutralized; therefore, we compared the mean standard deviation (SD) for the five ELP values, calculated at 2 months after surgery, between the two groups. Furthermore, on the basis of a report by Eom et al. [5], who reported that the root mean square (RMS) of the change in ELP at each follow-up visit (ELP_RMS_) could determine the axial position stability of IOLs more precisely than the mean ELP could; we calculated ELP_RMS_ for each group at 2 months after surgery using the following formula:

(1) $$\begin{array}{c} {\text{ELP}}_{\text{RMS}}=\sqrt{\frac{{\left({\text{ELP}}_{3\text{D}}-{\text{ELP}}_{1\text{D}}\right)}^{2}+{\left({\text{ELP}}_{1\text{W}}-{\text{ELP}}_{3\text{D}}\right)}^{2}+{\left({\text{ELP}}_{1\text{M}}-{\text{ELP}}_{1\text{W}}\right)}^{2}+{\left({\text{ELP}}_{2\text{M}}-{\text{ELP}}_{1\text{M}}\right)}^{2}}{4}}. \end{array}$$

Here, 1D, 3D, 1W, 1M, and 2M represent 1 day, 3 days, 1 week, 1 month, and 2 months after surgery, respectively.

### 2.5. Visual Acuity and Postoperative Refraction Error

The postoperative BCVA was recorded in logMAR units, and autorefraction (RK-F2, Canon Inc., Tokyo, Japan) was performed at each visit to evaluate the surgical outcomes. To demonstrate the discrepancy between the preoperatively calculated refraction (Lenstar LS900®) and the postoperative refraction at each time point, we calculated the postoperative refraction error (PRE) as the postoperative spherical equivalent (SE) minus the preoperative SE (SE = sphere + cylinder/2).

### 2.6. Specular Microscopy

The preoperative central corneal endothelial cell density (ECD), coefficient of variation (CV), and percentage of hexagonal cells were measured with a specular microscope (SP-9000, Konan Medical, Nishinomiya, Hyogo, Japan). To rule out the effect of anterior capsule polishing using a bimanual irrigation/aspiration system on corneal endothelial cell loss, the central corneal ECD, CV, and hexagonality were measured again at 1 and 2 months after surgery.

### 2.7. Statistical Analysis

The number of participants required to achieve a statistical power of 80% at a level of significance of 0.05 was 27. This sample size calculation was performed using the statistical freeware G∗Power (version 3.1.9.2) [16]. Data are reported as means and standard deviations. All statistical analyses were performed using SPSS for Windows (version 22.0, SPSS Inc.). Data normality was assessed using the Kolmogorov–Smirnov test. For intereye comparisons of variables with a normal distribution, a paired *t-*test was used. A *p* value of <0.05 was considered statistically significant.

## 3. Results

Sixty eyes of 30 patients were included, and nine patients (33.3%) were men. All patients underwent uneventful surgeries with no intraoperative or postoperative complications and returned on time for measurements. The mean age of patients was 74.1 ± 8.9 years (range, 51 to 88 years).  Table 1 shows the preoperative data, including BCVA; nuclear opalescence, cortical cataract, and posterior subcapsular cataract grades; ACD; AL; IOL power; and the predicted refraction, for both groups. There were no significant differences between groups in any of these parameters (*p* > 0.05).

**Figure 2:**
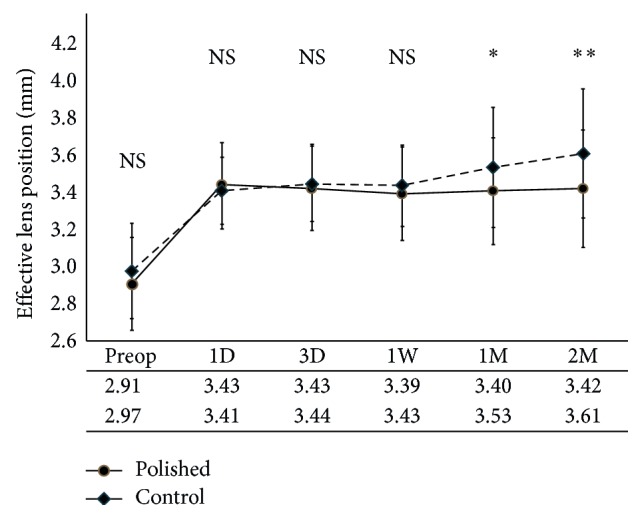
The mean effective lens position (ELP) at different visits in eyes with (polishing group) or without (control group) anterior capsule polishing during cataract surgery. There are no significant differences between groups in the mean ELP at 1 day, 3 days, and 1 week after surgery. The mean ELP at 1 and 2 months after surgery is significantly different (*p*=0.026 and 0.004, resp.).

Figure 2 shows the mean ELP at the different visits for each group. The mean ELP showed no significant difference between groups at 1 day, 3 days, and 1 week after surgery. However, significant differences were observed in the mean ELP at 1 and 2 months after surgery (*p*=0.026 and 0.004, resp.). The mean SD for the five ELP values was significantly smaller for the polishing group (0.087 ± 0.093 mm) than that for the control group (0.159 ± 0.138 mm; *p*=0.001). ELP_RMS_ was also significantly smaller for the polishing group (0.124 ± 0.034 mm) than that for the control group (0.246 ± 0.038 mm; *p*=0.047; Table 2).

PRE was 0.29 ± 0.74, 0.23 ± 1.33, −0.03 ± 1.23, 0.02 ± 0.78, and 0.06 ± 0.60 D at 1 day, 3 days, 1 week, 1 month, and 2 months after surgery, respectively, in the polishing group. For the control group, these values were 0.27 ± 0.43, 0.25 ± 0.93, 0.13 ± 0.66, 0.23 ± 1.01, and 0.27 ± 0.93 D, respectively. There were no significant differences between the two groups (1 day: *p*=0.901, 3 days: *p*=0.966, 1 week: *p*=0.524, 1 month: *p*=0.314, 2 months: *p*=0.309).

BCVA at 1 day, 3 days, 1 week, 1 month, and 2 months after surgery was 0.08 ± 0.10, 0.06 ± 0.09, 0.03 ± 0.06, 0.03 ± 0.06, and 0.02 ± 0.05, respectively, in the polishing group. These values were 0.07 ± 0.10, 0.05 ± 0.08, 0.03 ± 0.06, 0.02 ± 0.06, and 0.01 ± 0.04, respectively, in the control group. There were no significant differences between groups (1 day: *p*=0.489, 3 days: *p*=0.662, 1 week: *p*=0.573, 1 month: *p*=0.745, and 2 months: *p*=0.573).

Table 3 shows the preoperative and postoperative ECD, CV, and hexagonality values for both groups. There were no significant differences in any variable between the two groups (*p* > 0.05).

## 4. Discussion

There are many studies concerning the influence of anterior capsule polishing during surgeries involving the implantation of different IOLs on anterior capsule opacification (ACO), posterior capsule opacification (PCO), and the size of the capsulorhexis opening [11, 13, 14, 17]. However, few studies have assessed the effects of anterior capsule polishing on ELP or the axial position stability of IOL. Gao et al. [17] reported that anterior capsule polishing improved the axial position stability of the AcrySof IQ SN60WF IOL at 6 months after surgery, although no significant difference (no more than 0.06 mm) was detected in the mean ELP measured using anterior segment optical coherence tomography (AS-OCT) between the polishing and control groups. In our study, however, there were significant differences in the mean ELP at 1 month and 2 months after surgery between the polishing and control groups. We found that eyes that did not receive intraoperative anterior capsule polishing demonstrated a tendency for backward IOL movement by approximately 0.2 mm, with a concurrent hyperopic shift in refraction of approximately 0.2 D, at 2 months after surgery. Although this subtle change did not affect BCVA, the hyperopic shift after cataract surgery may be a problem, particularly for patients receiving refraction-correcting IOLs.

The mechanism underlying backward IOL movement in the absence of anterior capsule polishing remains uncertain. After cataract surgery, residual anterior LECs undergo fibrous metaplasia after contact with the anterior IOL surface [18]. These metaplastic LECs consist of *α*-smooth muscle actin elements that lead to anterior capsule contraction, constriction of the capsulorhexis and ACO [9]. This enhanced contractile force of the intrinsically elastic capsular membrane may result in a more potent centripetal capsular force and increased tensile force of the zonules attached to the capsule, eventually leading to backward shift of the IOL-capsule complex. In the present study, the number of eyes with anterior capsule fibrosis, which was measured using slit-lamp examination under pupil dilation at the last follow-up visit (2 months), was significantly lower in the polishing group (two eyes) than in the control group (nine eyes). Furthermore, anterior LECs migrate to the posterior capsule and result in fibrotic PCO as well as posterior capsule wrinkling [19]. In fact, there was no detectable posterior capsule fibrosis in any eye in the present study, probably because of the relatively short follow-up period. Constriction of the anterior and posterior capsules may synergistically result in a shift in the axial position of the IOL-capsule complex [8]. Therefore, the axial position of IOLs may remain more stable after the elimination of LECs under the anterior capsule during cataract surgery.

The axial position stability is also related to the mechanical characteristics of IOL, such as the design and material of the optic/haptic, optic-haptic angulation, and diameter [7]. In the present study, we used a single type of IOL, the Sensar AAB00 IOL, which has a hydrophobic acrylic optic with a diameter of 6.0 mm, an overall length of 13.0 mm, haptics of the same material, and no haptic angulation (0°). This hydrophobic, spherical, one-piece IOL demonstrated relatively little axial shift as well as a small refraction error and minimal BCVA changes during the follow-up period. This finding is consistent with those in previous studies showing that one-piece, hydrophobic, acrylic IOLs display little axial movement associated with stable postoperative refraction [5, 20]. The longer the overall length of IOL, the more it thrusts the equator of the capsule and the more stable is its axial position [21]. Furthermore, nonangulated IOLs show lesser postoperative axial movement than do angulated IOLs; a sharp optic edge design to prevent PCO has little influence on the axial position stability of IOL [11]. Not proven in the Sensar IOL, hydrophobic acrylic has bioadhesive characteristics that enhance the adhesion of IOL to the capsular bag and leads to less proliferation of LECs and anterior and posterior capsule fibrosis, with successive alleviation of capsule contraction and optic movement [22, 23]. Several studies have reported that anterior capsule contraction was significantly greater after hydrophilic IOL implantation than after hydrophobic IOL implantation [24–26]. We expect that more definite results may be found in further studies using hydrophilic acrylic IOLs instead of hydrophobic acrylic IOLs.

The main limitation of this study is the short follow-up period of 2 months after surgery, during which few instances of ACO, as well as no PCO, were detected. Moreover, our study population size is somewhat small despite the high statistical power of the paired *t*-test that we used. A larger population size, as well as follow-up periods that extend beyond 1 year (when most capsular events have already occurred), are necessary in future studies. Furthermore, we measured postoperative ELP using A-scan ultrasound images, not AS-OCT. Nemeth et al. [27] reported that the repeatability (intraobserver CV) of AS-OCT by two observers (0.8% and 1.9%) was superior to that of immersion A-scans (6.4% and 8.5%), whereas the reproducibility (interobserver CV) was comparable between the two modalities (0.23% and 0.88%, resp.). However, AS-OCT was not available in our clinic; therefore, we repeated the measurements five times and used the average value to improve the repeatability. Finally, no significant discrepancy in the postoperative visual acuity and refraction error was detected between the polishing and control groups. The improvement in the axial position stability by anterior capsule polishing may not be noticed by patients, although it is imperative in refractive cataract surgery, which requires a remarkably high level of surgical accuracy. Larger study samples with the implantation of various refraction-correcting IOLs are necessary to clarify this aspect.

## 5. Conclusions

In the present study, we polished the anterior capsule to eliminate residual LECs using a bimanual irrigation/aspiration system to assess the effects of this procedure on the axial position stability of hydrophobic, spherical, one-piece IOLs. We found that anterior capsule polishing enhanced the axial position stability of IOL without any complications. Without anterior capsule polishing, IOL tended to move backwards by approximately 0.2 mm, with a concurrent hyperopic shift in refraction of approximately 0.2 D, at 2 months after surgery. With the advancement of various refraction-correcting IOLs, the axial position stability becomes an important aspect. Therefore, anterior capsule polishing using a bimanual irrigation/aspiration system during cataract surgery may be considered a useful procedure, particularly for eyes receiving refraction-correcting IOLs.

## Figures and Tables

**Table 1:** Preoperative data for eyes with (polishing group) or without (control group) anterior capsule polishing during cataract surgery.

	Polishing group	Control group	*p* value^*∗*^
Best-corrected visual acuity (logMAR)	0.61 ± 0.37	0.64 ± 0.37	0.695
Nuclear opalescence grade	3.03 ± 0.85	2.80 ± 0.85	0.335
Cortical cataract grade	2.27 ± 0.83	2.13 ± 0.82	0.489
Posterior subcapsular cataract grade	1.97 ± 0.85	2.10 ± 0.76	0.382
Anterior chamber depth (mm)	2.91 ± 0.25	2.97 ± 0.26	0.203
Axial length (mm)	22.92 ± 0.70	23.04 ± 0.75	0.392
Intraocular lens power (D)	22.25 ± 2.06	21.77 ± 2.21	0.306
Predicted refraction (D)	−0.15 ± 0.45	−0.20 ± 0.50	0.284

Values are presented as means ± standard deviations. ^*∗*^Statistical analysis was performed by using a paired *t*-test.

**Table 2:** The postoperative effective lens position in eyes with (polishing group) or without (control group) anterior capsule polishing during cataract surgery.

Group	Mean ELP (mm)					Mean SD (mm)	Mean ELP_RMS_ (mm)
	1 day	3 days	1 week	1 month	2 months		
Polishing	3.43 ± 0.23	3.43 ± 0.23	3.39 ± 0.25	3.40 ± 0.29	3.42 ± 0.32	0.087 ± 0.093	0.124 ± 0 .034
Control	3.41 ± 0.18	3.44 ± 0.20	3.43 ± 0.22	3.53 ± 0.32	3.61 ± 0.35	0.159 ± 0.138	0.246 ± 0.038
*p* value^*∗*^	0.499	0.586	0.320	0.026	0.004	0.001	0.047

ELP, effective lens position; SD, standard deviation for ELP values obtained at the five time points; ELP_RMS_, root mean square of the change in ELP at each time point. ^*∗*^Statistical analysis was performed by using a paired *t*-test.

**Table 3:** Pre-/postoperative (1 and 2 months after surgery) specular microscopy findings for eyes with (polishing group) or without (control group) anterior capsule polishing during cataract surgery.

Group	Mean ECD (cells/mm^2^)			Mean CV			Mean hexagonality (%)		
	Preoperative	1 month	2 months	Preoperative	1 month	2 months	Preoperative	1 month	2 months
Polishing	2540.9 ± 426.7	2357.6 ± 497.9	2279.4 ± 575.3	28.83 ± 5.77	31.77 ± 5.82	32.10 ± 6.00	65.30 ± 6.29	64.03 ± 6.28	63.87 ± 6.79
Control	2430.9 ± 361.8	2323.8 ± 302.1	2259.2 ± 454.9	28.13 ± 5.69	29.63 ± 5.88	30.67 ± 6.96	66.07 ± 5.97	64.80 ± 6.60	64.40 ± 6.54
*p* value^*∗*^	0.541	0.066	0.401	0.254	0.110	0.164	0.217	0.573	0.638

ECD, endothelial cell density; CV, coefficient of variation. ^*∗*^Statistical analysis was performed by using a paired *t*-test.

## Data Availability

The datasets used to support this study are currently under embargo, while the research findings are commercialized. Requests for data at 12 months after initial publication will be considered by the corresponding author.
